# Supplementary material for: Effectiveness and Safety of Progressive Loading–Motion Style Acupuncture Treatment for Acute Low Back Pain after Traffic Accidents: A Randomized Controlled Trial
Source: Healthcare (Basel). 2023 Nov 10;11(22):2939. doi: 10.3390/healthcare11222939 (PMC10671479; doi:10.3390/healthcare11222939)
Supplement: Supplementary file 1 [file healthcare-11-02939-s001.zip › healthcare-2673726-supplementary.pdf]

Supplementary Table S1. Details of motion style acupuncture treatment (MSAT) and traditional acupuncture treatment based on the STRICTA 2010 checklist.

Item	Detail
**1. Acupuncture rationale**	**1a) Style of acupuncture:**<br>MSAT: 3 days of motion style acupuncture + traditional Korean acupuncture<br>Control: traditional Korean acupuncture
	**1b) Reasoning for treatment provided, based on historical context, sources in the literature, and/or consensus methods, with references where appropriate:**<br>MSAT: used by Korean medicine doctors (1, 2) and based on the consensus of a group of clinical experts<br>Traditional Korean: based on the textbook of acupuncture and commonly used by Korean medicine doctors
	**1c) Extent to which treatment was varied:**<br>MSAT: each treatment is performed for about 10 minutes, but the degree and frequency of movement can be adjusted at the discretion of the doctor<br>Traditional Korean: 6-12 needles among essential and optional acupoints
**2. Details of needling**	**2a) Number of needle insertions per subject per session:**<br>MSAT: 6 acupoints/traditional Korean: 6-12 acupoints
	**2b) Names (or location if no standard name) of points used (uni/bilateral):**<br>MSAT: GV4 or GV3, LR2 bilateral<br>Traditional Korean: essential—BL24, BL25, BL26; bilateral/optional—BL40, SP6, BL23 bilateral
	**2c) Depth of insertion, based on a specified unit of measurement, or on a particular tissue level:**<br>MSAT: 15-20 mm at GV4 or GV3, 10-15 mm at LR2<br>Traditional Korean: based on the physician's judgment
	**2d) Response sought (e.g., de qi or muscle twitch response):**<br>MSAT: none; traditional Korean: de qi
	**2e) Needle stimulation (e.g., manual, electrical):**<br>MSAT: manual stimulation during movement<br>Traditional Korean: electrical stimulation
	**2f) Needle retention time:**<br>MSAT: about 10 minutes; traditional Korean: 15 minutes
	**2g) Needle type (diameter, length, and manufacturer or material):**<br>0.30 * 40 mm, stainless steel, Dongbang medical, Korea
**3. Treatment regimen**	**3a) Number of treatment sessions**<br>MSAT: 3 sessions; traditional Korean: 8-26 sessions
	**3b) Frequency and duration of treatment sessions:**<br>MSAT: once a day for 3 days; traditional Korean: twice a day for 5–13 days
**4. Other components of treatment**	**4a) Details of other interventions administered to the acupuncture group (e.g., moxibustion, cupping, herbs, exercises, lifestyle advice):**<br>Integrative Korean medicine treatment, such as acupuncture, pharmacopuncture, chuna manual therapy, and herbal medicine
	**4b) Setting and context of treatment, including instructions to practitioners, and information and explanations to patients:**<br>None in particular

**5. Practitioner background**	**5) Description of participating acupuncturists (qualification or professional affiliation, years in acupuncture practice, other relevant experience):**<br>Korean medicine doctor with at least 3 years of clinical experience and affiliated with Bucheon Jaseng hospital.
**6. Control or comparator interventions**	**6a) Rationale for the control or comparator in the context of the research question, with sources that justify this choice:**<br>For the purpose of the study evaluating the effectiveness of additional treatment with MSAT in traditional Korean medicine treatment, the control was defined as a group that received integrative Korean medicine treatment only<br>**6b) Precise description of the control or comparator. If sham acupuncture or any other type of acupuncture-like control is used, provide details as for Items 1 to 3 above:**<br>Integrative Korean medicine treatment such as acupuncture, pharmacopuncture, chuna manual therapy, herbal medicine, etc. Details related to acupuncture are mentioned for items 1–3

Abbreviations: MSAT, motion style acupuncture treatment.

Supplementary Table S2. Study schedule and measurements at each visit.

	Study period						
	Screening	Active treatment				Follow up	
Visit	Day[1] 1 (V1)	Day 2 (V2)	Day 3 (V3)	Day 4 (V4)	D/C (V5)	1 month[2] (V6)	3 month (V7)
**Enrollment**							
Informed consent form	○						
Confirm suitability for study	○						
Block randomization	○						
Credibility and Expectancy Questionnaire	○						
History (post-TA neck pain and other)	○						
**Intervention**							
MSAT+IKMT	△ (only IKMT)	○	○	○	△ (only IKMT)		
IKMT	○	○	○	○	○		
**Assessment**							
Check symptoms and Med change		○	○	○	○	○	○
NRS of low back pain	○	○*	○*	○*	○	○	○
NRS of leg pain	○	○♀	○♀	○♀	○	○	○
VAS of low back pain		○†	○†	○†	○		
VAS of leg pain		○¥	○¥	○¥	○		
ODI		○		○♭	○	○	○
ROM of lumbar		○¶	○¶	○¶	○		
EQ-5D		○		○♭	○	○	○
EQ-VAS		○		○♭	○		
SF-12		○		○♭	○	○	○
PGIC		○		○♭	○	○	○
CL-5-K		○		○♭	○	○	○
Adverse events		○	○	○	○	○	○
Drug consumption	○	○	○	○	○	○	○

Notes: [1] Days from admission, [2] months from discharge, * additional NRS of low back pain after treatment, ♀ additional NRS of leg pain after treatment, † additional VAS of low back pain after treatment, ¥ additional VAS of leg pain after treatment, ¶ additional ROM of lumbar after treatment, ♭ investigation was performed only when the 4th day of hospitalization (V4) and discharge date were the same. Abbreviations: V, visit; MSAT, motion style acupuncture treatment; IKMT, integrative Korean medicine treatment; NRS, numeric rating scale; VAS, visual analogue scale; ODI, Oswestry disability index; ROM, range of motion; EQ-5D, Quality of Life EuroQol 5-Dimension; EQ-VAS, EuroQol visual analogue scale; SF-12, Short Form-12 health survey version 2; PGIC, patient global impression of change; PCL-5-K, Post-Traumatic Stress Disorder Checklist for DSM-5.

Supplementary Table S3. Comparison of disability outcomes at each measuring point between the PL-MSAT and control groups.

		V2-2	V3-1	V3-2	V4-1	V4-2	D/C	1M	3M
ODI score	PL-MSAT group						27.02 (24.83–29.22)	21.33 (17.92–24.74)	16.17 (13.51–18.83)
	Control group						27.84 (25.72–29.96)	20.68 (17.28–24.09)	14.29 (11.64–16.93)
	Difference						0.81 (–2.32 to 3.95)	–0.65 (–5.53 to 4.23)	–1.88 (–5.67 to 1.91)
	P value						0.608	0.792	0.327
ROM flexion	PL-MSAT group	73.04 (71.30–74.79)	74.69 (72.69–76.68)	77.41 (75.35–79.47)	78.26 (75.99–80.53)	81.83 (79.87–83.80)	86.33 (84.54–88.12)		
	Control group	69.47 (67.74–71.20)	71.77 (69.79–73.76)	71.95 (69.91–73.98)	76.10 (73.80–78.41)	76.52 (74.53–78.51)	81.60 (79.84–83.35)		
	Difference	–3.58 (–6.06 to –1.09)	–2.91 (–5.75 to –0.08)	–5.46 (–8.40 to –2.53)	–2.16 (–5.38 to 1.07)	–5.31 (–8.15 to –2.48)	–4.73 (–7.27 to –2.19)		
	P value	0.005**	0.044*	<0.001***	0.187	<0.001***	<0.001***		
ROM extension	PL-MSAT group	12.73 (12.01–13.46)	13.41 (12.60–14.22)	14.39 (13.48–15.30)	14.84 (13.92–15.76)	15.34 (14.43–16.25)	17.54 (16.70–18.37)		
	Control group	10.75 (10.03–11.47)	11.07 (10.28–11.87)	11.45 (10.55–12.34)	12.78 (11.88–13.68)	13.25 (12.36–14.14)	14.95 (14.13–15.78)		
	Difference	–1.98 (–3.02 to –0.95)	–2.33 (–3.48 to –1.19)	–2.94 (–4.24 to –1.65)	–2.06 (–3.36 to –0.76)	–2.09 (–3.39 to –0.80)	–2.58 (–3.77 to –1.39)		
	P value	<0.001***	<0.001***	<0.001***	0.002**	0.002**	<0.001***		
ROM lateral flexion (Rt.)	PL-MSAT group	19.17 (18.64–19.70)	20.40 (19.37–21.43)	21.71 (20.56–22.86)	23.14 (21.98–24.30)	24.22 (22.97–25.47)	27.51 (26.55–28.46)		
	Control group	18.46 (17.93–18.99)	20.41 (19.36–21.45)	20.72 (19.59–21.86)	21.94 (20.73–23.16)	22.26 (20.90–23.61)	24.29 (23.35–25.22)		
	Difference	–0.71 (–1.46 to 0.05)	0.00 (–1.48 to 1.49)	–0.99 (–2.64 to 0.67)	–1.20 (–2.92 to 0.52)	–1.96 (–3.85 to –0.08)	–3.22 (–4.58 to –1.86)		
	P value	0.066	0.995	0.238	0.169	0.042*	<0.001***		
ROM lateral flexion (Lt.)	PL-MSAT group	18.98 (18.35–19.61)	20.19 (19.03–21.36)	21.68 (20.42–22.94)	23.02 (21.83–24.22)	24.38 (23.11–25.65)	27.63 (26.64–28.62)		
	Control group	17.57 (16.95–18.20)	19.94 (18.78–21.10)	20.30 (19.04–21.55)	22.07 (20.84–23.31)	22.07 (20.74–23.40)	24.16 (23.19–25.13)		
	Difference	–1.40 (–2.30 to –0.51)	–0.25 (–1.92 to 1.42)	–1.38 (–3.19 to 0.43)	–0.95 (–2.71 to 0.81)	–2.31 (–4.21 to –0.41)	–3.47 (–4.88 to –2.06)		
	P value	0.003**	0.767	0.133	0.287	0.017*	<0.001***		
ROM rotation (Rt.)	PL-MSAT group	31.57 (30.78–32.36)	33.42 (32.16–34.68)	34.69 (33.39–36.00)	36.20 (34.90–37.51)	37.41 (36.00–38.82)	41.07 (39.79–42.35)		
	Control group	29.93 (29.15–30.71)	32.56 (31.28–33.84)	33.03 (31.74–34.32)	35.31 (33.97–36.66)	36.57 (35.09–38.06)	38.57 (37.30–39.83)		
	Difference	–1.64 (–2.77 to –0.52)	–0.86 (–2.68 to 0.96)	–1.66 (–3.55 to 0.22)	–0.89 (–2.82 to 1.04)	–0.84 (–2.92 to 1.25)	–2.51 (–4.34 to –0.67)		
	P value	0.005**	0.35	0.083	0.363	0.427	0.008**		
ROM rotation (Lt.)	PL-MSAT group	32.22 (31.48–32.95)	33.68 (32.38–34.97)	35.04 (33.73–36.34)	36.69 (35.42–37.95)	37.59 (36.17–39.01)	41.38 (40.18–42.59)		
	Control group	30.77 (30.04–31.49)	33.05 (31.75–34.35)	33.54 (32.22–34.85)	35.36 (34.06–36.65)	36.46 (34.95–37.97)	38.56 (37.37–39.74)		
	Difference	–1.45 (–2.49 to –0.41)	–0.63 (–2.48 to 1.23)	–1.50 (–3.41 to 0.41)	–1.33 (–3.18 to 0.52)	–1.13 (–3.20 to 0.94)	–2.83 (–4.55 to –1.11)		
	P value	0.007**	0.506	0.122	0.156	0.282	0.001**		

Notes: Outcomes were analyzed according to the intention-to-treat principle, and missing data were imputed with multiple imputations. The dashes indicate that outcome measurements were not administered. The outcome measurements at the 14-day follow-up were excluded because they were similar to the discharge outcomes. The mean lengths of stay in the PL-MSAT and control groups were 8.73 ± 3.84 and 8.41 ± 3.91

days, respectively. Five and six participants in the PL-MSAT and control groups, respectively, were discharged before treatment completion. The differences between the PL-MSAT and control groups are shown as the mean and 95% confidential interval. Analysis of covariance was performed to calculate the differences and p-values. The covariates included each baseline of each outcome and PCL-5-K. The values are presented with 95% confidence interval. * P < 0.05; ** P < 0.01; *** P < 0.001. **Abbreviations:** V, visit; D/C, discharge; M, month; PL-MSAT, progressive loading–motion style acupuncture treatment; ODI, Oswestry disability index; ROM, range of motion; Rt., right; Lt., left.

Supplementary Table S4. Comparison of quality-of-life outcomes at each measuring point between the PL-MSAT and control groups.

		V2-2	V3-1	V3-2	V4-1	V4-2	D/C	1M	3M
EQ-5D-5L	PL-MSAT group						0.77 (0.74–0.80)	0.80 (0.78–0.83)	0.84 (0.82–0.87)
	Control group						0.79 (0.76–0.81)	0.84 (0.81–0.86)	0.86 (0.84–0.89)
	Difference						0.02 (−0.02 to 0.06)	0.03 (0.00–0.07)	0.02 (−0.02 to 0.06)
	P value						0.344	0.08	0.273
PCS	PL-MSAT group						39.50 (37.74–41.26)	43.74 (41.88–45.59)	47.38 (45.45–49.31)
	Control group						41.91 (40.19–43.62)	45.23 (43.37–47.09)	47.97 (46.03–49.91)
	Difference						2.41 (−0.10 to 4.92)	1.50 (−1.17 to 4.17)	0.60 (−2.18 to 3.37)
	P value						0.06	0.269	0.671
MCS	PL-MSAT group						46.99 (44.61–49.37)	51.06 (48.55–53.56)	52.84 (50.70–54.99)
	Control group						45.29 (42.97–47.62)	51.44 (48.95–53.92)	52.36 (50.23–54.48)
	Difference						−1.70 (−5.09 to 1.70)	0.38 (−3.21 to 3.97)	−0.49 (−3.55 to 2.57)
	P value						0.324	0.833	0.753

Notes: Outcomes were analyzed according to the intention-to-treat principle, and missing data were imputed with multiple imputations. The dashes indicate that outcome measurements were not administered. The outcome measurements at the 14-day follow-up were excluded because they were similar to the discharge outcomes. The mean lengths of stay in the PL-MSAT and control groups were 8.73 ± 3.84 and 8.41 ± 3.91 days, respectively. Five and six participants in the PL-MSAT and control groups, respectively, were discharged before treatment completion. The differences between the PL-MSAT and control groups are shown as the mean and 95% confidential interval. Analysis of covariance was performed to calculate the differences and p-values. The covariates included each baseline of each outcome and PCL-5-K. The values are presented with 95% confidence interval. * P < 0.05; ** P < 0.01; *** P < 0.001. **Abbreviations:** V, visit; D/C, discharge; M, month; PL-MSAT, progressive loading–motion style acupuncture treatment; EQ-5D, Quality of Life EuroQol 5-Dimension; PCS, physical component summary; MCS, mental component summary.

Supplementary Table S5. Comparison of PGIC at each measuring point between the PL-MSAT and control groups.

		V2-2	V3-1	V3-2	V4-1	V4-2	D/C	1M	3M
PGIC	PL-MSAT group	3.36 (3.19–3.53)					2.45 (2.26–2.65)	2.38 (2.15–2.61)	2.38 (2.14–2.62)
	Control group	3.75 (3.58–3.92)					2.40 (2.20–2.59)	2.19 (1.95–2.43)	1.98 (1.74–2.22)
	Difference	−0.39 (−0.63 to −0.15)					0.06 (−0.22 to 0.34)	0.19 (−0.14 to 0.52)	0.40 (0.06–0.75)
	P value	0.002**					0.686	0.261	0.023*

Notes: Outcomes were analyzed according to the intention-to-treat principle, and missing data were imputed with multiple imputations. The dashes indicate that outcome measurements were not administered. The outcome measurements at the 14-day follow-up were excluded because they were similar to the discharge outcomes. The mean lengths of stay in the PL-MSAT and control groups were 8.73 ± 3.84 and 8.41 ± 3.91 days, respectively. Five and six participants in the PL-MSAT and control groups, respectively, were discharged before treatment completion. The differences between PL-MSAT and control groups are shown as the mean and 95% confidential interval. Analysis of covariance was performed to calculate the differences and p-values. The covariates included each baseline of each outcome and PCL-5-K. The values are presented with 95% confidence interval. * P < 0.05; ** P < 0.01; *** P < 0.001. **Abbreviations:** V, visit; D/C, discharge; M, month; PL-MSAT, progressive loading–motion style acupuncture treatment; PGIC, patient global impression of change.

Supplementary Table S6. Comparison of PCL-5-K at each measuring point between the PL-MSAT and control groups.

		V2-2	V3-1	V3-2	V4-1	V4-2	D/C	1M	3M
PCL-5-K	PL-MSAT group						16.46 (13.87–19.06)	11.57 (9.06–14.09)	9.20 (6.81–11.59)
	Control group						16.23 (13.74–18.72)	10.85 (8.39–13.32)	8.61 (6.27–10.94)
	Difference						–0.23 (–3.89 to 3.42)	–0.72 (–4.29 to 2.85)	–0.59 (–3.98 to 2.79)
	P value						0.9	0.69	0.728

Notes: Outcomes were analyzed according to the intention-to-treat principle, and missing data were imputed with multiple imputations. The dashes indicate that outcome measurements were not administered. The outcome measurements at the 14-day follow-up were excluded because they were similar to the discharge outcomes. The mean lengths of stay in the PL-MSAT and control groups were 8.73 ± 3.84 and 8.41 ± 3.91 days, respectively. Five and six participants in the PL-MSAT and control groups, respectively, were discharged before treatment completion. The differences between the PL-MSAT and control groups are shown as the mean and 95% confidential interval. Analysis of covariance was performed to calculate the differences and p-values. The covariates included each baseline of each outcome and PCL-5-K. The values are presented with 95% confidence interval. * P < 0.05; ** P < 0.01; *** P < 0.001. **Abbreviations:** V, visit; D/C, discharge; M, month; PL-MSAT, progressive loading–motion style acupuncture treatment; PCL-5-K, Post-Traumatic Stress Disorder Checklist for DSM-5.
